# Breast Cancer in Sub-Saharan Africa

**DOI:** 10.1200/JGO.2016.008433

**Published:** 2017-03-27

**Authors:** Edward L. Trimble

**Affiliations:** **Edward L. Trimble**, National Cancer Institute, Bethesda, MD

In this issue, Vanderpuye et al^1^ report an initial survey of members of the African Organization for Research and Treatment of Cancer who reside in sub-Saharan Africa about breast cancer management. As the authors note, the sample size is small—namely, 20 respondents from 19 facilities across an area with a population of more than 950 million people. The disciplines represented include those generally involved with breast cancer management: surgery, medical oncology, clinical oncology, radiation therapy, pathology, and nursing. The respondents noted major issues that included late presentation and limited access to imaging, pathology, radiation therapy, sentinel lymph node assessment, chemotherapy, hormonal therapy, and multidisciplinary care. In addition, they noted that many women who have breast cancer face financial constraints in payment for care.

The International Agency for Research on Cancer has estimated that the burden of breast cancer in sub-Saharan Africa in 2012 was approximately 100,000 occurrences per year and 49,000 deaths per year.^2^ In most countries of sub-Saharan Africa, breast cancer is the second most common cancer type among women, after cervical cancer. The challenges laid out in the report by Vanderpuye et al^1^ appear at first glance almost insurmountable. Nonetheless, under the leadership of Benjamin Anderson and his colleagues, the Breast Global Health Initiative has developed a thoughtful set of evidence-based, resource-stratified guidelines to screen, diagnose, and treat breast cancer.^3^ More recently, the National Comprehensive Cancer Network has undertaken an expansion of its guidelines to include a resource-stratified approach for other common cancers, including cervical and colon cancers.^4^

Some innovative approaches to diagnose breast cancer and strengthen health systems also show promise for cancer care in low-resource settings. These include the use of novel, low-cost hand-held imaging devices for the evaluation of breast masses and novel techniques for the evaluation of estrogen-receptor and progesterone-receptor statuses on fine-needle aspiration samples. In addition, multiple efforts are underway to improve access to pathologic diagnosis in sub-Saharan Africa through expanded training, telemedicine, and other approaches.^5^ The WHO is working with countries in sub-Saharan Africa and elsewhere to address the pervasive issue of substandard, spurious, falsely labeled, falsified, and counterfeit drugs, including oncologic agents. Pharmaceutical companies have pledged to improve access to novel therapeutics in low-resource settings. Public-private partnerships have demonstrated some success to improve access to radiation therapy.

Nonetheless, this report by Vanderpuye et al^1^ underscores the importance of basic lessons in cancer control that we have learned during the past decades. Overcoming the stigma of cancer is critical, as are public awareness of breast health and the need for timely evaluation of breast masses. Access to timely and high-quality imaging and pathologic diagnosis remains essential. Multidisciplinary tumor boards should review management plans for individual patients. Care should be patient centered and include timely access to high-quality surgery, radiation therapy, chemotherapy, hormonal therapy, supportive care, survivorship care, and palliative care, as appropriate. There must be national strategies in place to educate and retain cancer specialists in the required disciplines, including surgery, radiation oncology/clinical oncology/medical oncology, pathology, imaging, nursing, radiation therapy, and allied health. Medical informatics should permit linkages of health records for patients with cancer to cancer registries and death registries. Finally, some combination of universal health care, private insurance, public insurance, and charitable care must ensure that patients with cancer and their families can afford the appropriate treatment.
